# The economic burden of pediatric gastroenteritis to Bolivian families: a cross-sectional study of correlates of catastrophic cost and overall cost burden

**DOI:** 10.1186/1471-2458-14-642

**Published:** 2014-06-24

**Authors:** Rachel M Burke, Emily R Smith, Rebecca Moritz Dahl, Paulina A Rebolledo, Maria del Carmen Calderón, Beatriz Cañipa, Edgar Chavez, Rolando Pinto, Luis Tamayo, Carlos Terán, Ángel Veizaga, Remy Zumaran, Volga Iñiguez, Juan S Leon

**Affiliations:** 1 Department of Epidemiology, Rollins School of Public Health, Emory University, Atlanta, GA, USA; 2 Laney Graduate School, Emory University, Atlanta, GA, USA; 3 Division of Infectious Diseases, Emory University School of Medicine, Atlanta, GA, USA; 4 Hospital Mario Ortiz Suárez, Santa Cruz, Bolivia; 5 Hospital Materno-Infantil, La Paz, Bolivia; 6 Hospital Boliviano Holandés, El Alto, Bolivia; 7 Hospital del Niño Manuel Ascencio Villarroel, Cochabamba, Bolivia; 8 Hospital del Niño, La Paz, Bolivia; 9 Centro Pediatría, Hospital Albina R. Patiño, Cochabamba, Bolivia; 10 Instituto de Biología Molecular y Biotecnología, Universidad Mayor de San Andrés, La Paz, Bolivia; 11 Hubert Department of Global Health, Rollins School of Public Health, Emory University, Atlanta, GA, USA; 12 Department of Environmental Health, Rollins School of Public Health, Emory University, Atlanta, GA, USA

**Keywords:** Pediatric gastroenteritis, Diarrhea, Societal costs, Health economics

## Abstract

**Background:**

Worldwide, acute gastroenteritis causes substantial morbidity and mortality in children less than five years of age. In Bolivia, which has one of the lower GDPs in South America, 16% of child deaths can be attributed to diarrhea, and the costs associated with diarrhea can weigh heavily on patient families. To address this need, the study goal was to identify predictors of cost burden (diarrhea-related costs incurred as a percentage of annual income) and catastrophic cost (cost burden ≥ 1% of annual household income).

**Methods:**

From 2007 to 2009, researchers interviewed caregivers (n = 1,107) of pediatric patients (<5 years old) seeking treatment for diarrhea in six Bolivian hospitals. Caregivers were surveyed on demographics, clinical symptoms, direct (e.g. medication, consult fees), and indirect (e.g. lost wages) costs. Multivariate regression models (n = 551) were used to assess relationships of covariates to the outcomes of cost burden (linear model) and catastrophic cost (logistic model).

**Results:**

We determined that cost burden and catastrophic cost shared the same significant (p < 0.05) predictors. In the logistic model that also controlled for child sex, child age, household size, rural residence, transportations taken to the current visit, whether the child presented with complications, and whether this was the child’s first episode of diarrhea, significant predictors of catastrophic cost included outpatient status (OR 0.16, 95% CI [0.07, 0.37]); seeking care at a private hospital (OR 4.12, 95% CI [2.30, 7.41]); having previously sought treatment for this diarrheal episode (OR 3.92, 95% CI [1.64, 9.35]); and the number of days the child had diarrhea prior to the current visit (OR 1.14, 95% CI [1.05, 1.24]).

**Conclusions:**

Our analysis highlights the economic impact of pediatric diarrhea from the familial perspective and provides insight into potential areas of intervention to reduce associated economic burden.

## Background

Worldwide, acute gastroenteritis causes substantial morbidity and mortality in children under five years of age, with 1.4 billion episodes and 1.7 to 3 million deaths each year [1]. Diarrhea accounts for 21% of all child deaths in low and middle income countries (LMIC) [2]. Gastroenteritis presents an economic burden to both healthcare systems and patient families [3-5]. Though various studies have attempted to quantify the costs associated with pediatric diarrhea from the state perspective [3-12], fewer studies have specifically examined the perspective of the patient’s family [8-10,13-16], despite the burden that these costs may represent. Though health insurance may cover some costs associated with pediatric diarrhea, patient families often still incur substantial “direct” (i.e., out-of-pocket) and “indirect” (i.e., lost income) expenses [16-19]. Studies have estimated average total familial costs (direct and indirect) per episode of hospitalized pediatric diarrhea ranging from US$19.86 in Kenya (2007USD [6]) to US$215.88 in Mexico (2003USD [11]). Direct costs, alone, for a pediatric diarrhea episode have ranged from US$12.89 per case in Brazil (2007USD [13]) to US$31.83 per case in Vietnam (2004USD [4]). In a low-resource setting, these costs can represent a large proportion of a family’s overall budget. In one study in India, direct costs incurred per diarrheal episode ranged 2.2 – 5.8% of the household’s annual income [14]. The ratio of total incurred costs for a single diarrheal episode as a percentage of annual family income, termed the “cost burden,” has been infrequently studied. Nonetheless, large cost burdens incurred from healthcare expenses can have a serious effect on a family’s overall current and future economic situation, especially for families already on the edge of poverty (reviewed in [20,21]). Healthcare costs that cause poverty (e.g. by forcing a family to spend money needed for food or other basic necessities) are termed “catastrophic” [22]. There is little consensus in the literature as to the exact calculations and cut-offs that are most appropriate for defining catastrophic costs [23,24]. Some advocate the use of a cut-off based on expenditures as a percentage of “capacity to pay” (non-subsistence spending) [22,25,26]. However, when detailed information on household expenditures is not available, the use of a cut-off based on cost burden may be useful. While a cost burden of 10% of annual income is often used to define “catastrophic cost” [24,27], lower limits can also be catastrophic for poor households [21,28]. For example, in a study of catastrophic healthcare costs in Thailand, a cost burden of 10% of monthly income was utilized, which is equivalent to approximately 1% of annual income [29].

Bolivia has one of the lower GDPs in South America (per-capita 2013 GDP US$5,500) [30] and has high rates of child mortality. As of 2012, for every 1,000 live births in Bolivia, 41 children die before age five, with an estimated 8% of these deaths from diarrhea [31]. The cumulative financial impact of these diarrheal episodes may be severe in this setting, where 30% of the population lives on under US$2 per day (2009 est.) [30]. Although Bolivia does have a health program (Seguro Universal Materno-Infantil, SUMI) that covers children under five, families must register to benefit from free care [32]. In addition, free treatment to registered families may not be enforced (e.g. oversight, intentionally), benefits are only available at public healthcare settings, and not all potential treatments are eligible for coverage. Further, if medications are out of stock at the treating facility, caregivers may need to purchase drugs from pharmacies where SUMI does not apply. Thus, Bolivian families may still incur substantial costs related to pediatric diarrhea.

The goal of this study was to characterize the financial effects and potential predictors of costs due to an episode of pediatric diarrhea on the caregivers of Bolivian children (under five) who sought treatment for diarrhea from 2007 through 2009, prior to full implementation of the vaccine against rotavirus (a major cause of pediatric diarrhea [33]) into the immunization schedule [34-36].

## Methods

This work was done in collaboration with, but independently from, the Rotavirus Surveillance Project of Bolivia.

### Sample population and recruitment

Caregivers of children suffering from diarrhea were recruited from 2007 to 2009 in outpatient clinics, hospital wards, and emergency rooms in six healthcare settings across four cities: Hospital Boliviano Holandés in El Alto, Hospital Materno-Infantil and Hospital del Niño in La Paz, Centro de Pediatría Albina R. de Patiño and Hospital Germán Urquidi in Cochabamba, and Hospital Mario Ortiz Suárez in Santa Cruz. All six hospitals were sentinel sites for rotavirus surveillance, located in the four major cities of Bolivia; all provide both inpatient and outpatient care, and are located primarily in urban or peri-urban environments (though do draw some patients from rural areas). The sample size of 1,107 was estimated from the World Health Organization (WHO) report “Guidelines for Estimating the Economic Burden of Diarrheal disease with focus on Assessing the Costs of Rotavirus Diarrhea” [17]. To achieve a 10% precision and 0.5 coefficient of variation per hospital, at least 49 records were collected from each hospital. Based on sufficient sample size for analysis and ability to compare across hospitals and cities, only inpatient (N = 254) and outpatient (N = 297) visits with valid income data were considered, while emergency department (N = 80) and pharmacy (N = 2) were excluded; inpatients from hospital Germán Urquidi and outpatients from Hospitals Materno-Infantil and del Niño were also excluded for this reason (n < 10).

Potentially eligible caregivers were identified by attending clinicians (inpatients) or by study staff (outpatients). Clinicians confirmed diagnosis of acute pediatric diarrhea (non-bloody) as well as any complications (e.g. dehydration) with which children presented. Study staff explained the study and obtained consent. Caregivers were eligible if they were at least 18 years old and were responsible for a child under five who was currently receiving treatment for acute diarrhea.

Prior to data collection, the study was approved by Emory University’s Institutional Review Board (Protocol IRB00004406) and the Bolivian National Bioethics Committee.

### Healthcare cost determination and definitions

Healthcare costs were obtained from caregivers via questionnaires administered by trained study staff (questionnaire available upon request). Caregivers reported any costs that they incurred out of pocket. As described above, caregivers may incur out-of-pocket expenses if they are not be enrolled in SUMI, or may intentionally or unintentionally be asked to pay for SUMI-eligible expenses. The interview guide was informed by the same WHO report described above [17]. Variables were defined and calculated as described in Table 1. Costs in Bolivianos (BOB) were converted into USD using the 2012 exchange rate of 1 BOB equal to US$0.146. Costs were rounded to the nearest US$0.50. The diagnosis of an acute diarrheal episode was confirmed by clinicians, and its duration was reported by the caregiver via questionnaire.

**Table 1:** Calculations and definitions of cost variables as used in analyses of cost burden and catastrophic cost for Bolivian children (n = 551) seeking treatment for acute diarrhea, 2007 – 2009

**Variable**	**Calculation**
Direct Medical Costs^1^	Sum of fees or costs associated with diagnostics, cost of medication, consultation fees, and any costs of previous treatment for this diarrheal episode.
Direct Non-Medical Costs^1^	Sum of costs of transportation to and from the appointment (and previous appointments for this diarrheal episode), food bought during the hospital visit (which caregivers perceived as otherwise unnecessary), extra diapers purchased during the visit (perceived as above a family’s standard supply), and childcare for the caregiver’s other children during the episode.
Total Direct Costs^1^	Sum of medical and non-medical direct costs.
Indirect Costs^1^	Sum of lost wages by caregiver and his or her spouse, based on the caregiver’s report of 1) salary for herself and her spouse, and 2) the number of days of work missed by herself and her spouse due to this diarrheal episode.
Total Incurred Costs^1^	Sum of Total Direct and Total Indirect Costs.
Annual Household Income	Sum of reported monthly incomes for the caregiver and spouse, multiplied by 12. Where caregiver salary was missing and the caregiver reported having no job or being a homemaker, caregiver salary was set to zero (n = 305). Where spousal salary was missing and the spouse was reported as jobless, spousal salary was set to zero (n = 16). Otherwise, missing salaries were left missing. If at least one parent reported a salary, then the household income equaled the value reported by the salary-generating parent. If both parents had missing salaries, then the household income was considered missing data and excluded from analysis.
Cost Burden^2^	Total Incurred Costs divided by the Annual Household Income and expressed as a percentage.
Catastrophic Cost^2^	Cost Burden greater than or equal to 1% of annual income, approximately 10% of monthly income. Research suggests that this is an appropriate cut-off for hardship, in low-income settings.

^1^Based on definitions from the WHO guidelines for assessing the cost burden of diarrhea [17].

^2^Consistent with definitions by Weraphong et al. [29].

### Data entry and database management

Trained staff collected all data. Two separate staff double-entered data using Epi Info (v. 3.4.3). The two databases were compared, and discrepancies logged and corrected according to a paper copy of the survey. A random sample of 5% of the records was checked against the original data to confirm a 100% match.

### Statistical methods

All data cleaning and analyses were completed in SAS version 9.3 (Cary, NC). We used Spearman correlation coefficients (continuous variables), chi-square tests (categorical variables), and parametric (Analysis of Variance [ANOVA]) and non-parametric (Kruskal Wallis) tests to assess relationships among cost burden, catastrophic cost, and potential predictors. Multivariate regression was used to examine relationships among potential predictors and log_10_ of cost burden (linear, transformed to meet normality assumptions) or catastrophic cost burden (logistic).

Potential predictors (including interactions) were selected based on *a priori* and data-driven criteria, as well as data availability. Collinearity was not present in linear or logistic models (logistic assessed via Collinearity Diagnostics Information Matrix macro for SAS [37]). To determine the final linear model, we performed backwards elimination of potential interaction terms and required all models to be hierarchically well formulated [38]. Potential interactions in logistic models were assessed via Likelihood Ratio Tests. Given that we are looking at a predictive model rather than a causal one, we did not assess confounding, but instead used the fully adjusted models as the final models. Tests of global fit (Wald, Likelihood Ratio, and score) were significant (p < 0.01).

## Results

### Characteristics of the study population

To characterize costs of pediatric diarrhea to Bolivian families, we recruited caregivers from three geographic areas: La Paz and El Alto, Cochabamba, and Santa Cruz (Table 2). Children were evenly split in gender, and less than 20% of caregivers reported rural residence. The majority of caregivers (91%) were the children’s mothers. Most reported being SUMI-registered, and the mean age of children was about one year. The mean family size was just under five members, and the mean monthly income was US$243, with 30% of respondents coming from households where both spouses contributed to income. However, income was not normally distributed (median US$190). Most caregivers reported having sought treatment at least once prior to the current visit (significantly more common among outpatients, p < 0.01), and the average caregiver used about one mode of transportation to arrive (e.g. one trip in bus or one shared taxi ride as opposed to one trip in bus and one shared taxi ride). Approximately half the children were outpatients, about half presented with at least one complication, and 35% were experiencing their first-ever episode of diarrhea. The mean time that the child had been ill with diarrhea prior to the current visit was five days. The mean time ill with diarrhea was not associated with monthly household income (p = 0.23) or with income per capita (p = 0.15) in bivariate analyses.

**Table 2:** Characteristics of the study population (n = 551) of Bolivian children seeking hospital care for diarrheal episodes, 2007 - 2009

**Characteristic**	**n**	***Frequency (Percent) or mean (SD)***	
*Demographics*			
Caregiver relationship to child	542		
Mother		493	(91.0)
Father		38	(7.0)
Other relative		11	(2.0)
Male child	550	302	(54.9)
Age of child (months)	524	12.6	(9.5)
Rural residence	458	81	(17.7)
SUMI^†^	313	266	(85.0)
Hospital (City)	536		
Del Niño (La Paz)		25	(4.7)
Materno-Infantil (La Paz)		25	(4.7)
Boliviano Holandés (El Alto)		61	(11.4)
Germán Urquidi (Cochabamba)		75	(14.0)
Albina Patiño (Cochabamba)^‡^		203	(37.9)
Mario Ortiz Suárez (Santa Cruz)		147	(27.4)
Number of people in household	524	4.7	(2.4)
Average monthly household income (US$)	551	242.50	(200.00)
Dual-income household	477	145	(30.4)
*Treatment-Seeking Behavior*			
Sought treatment at least once previously to current visit	467	384	(82.2)
Number of transportations taken to current visit	428	1.3	(0.5)
Number of days child had diarrhea prior to current visit	513	4.9	(7.4)
*Severity of illness*			
Child was an outpatient	551	297	(53.9)
Child presented with at least one complication^§^	548	280	(51.1)
Child’s first episode of diarrhea in their life	466	164	(35.2)

^†^Universal insurance program for Bolivian children <5 and pregnant women (covered up to 6 mo. post partum). ^‡^Private hospital. ^§^Complications defined as at least one of the following, as diagnosed by the attending physician: electrolyte disorder, electrolyte imbalance, hypokalemia, metabolic acidosis, anemia, malnutrition, acute respiratory infection, bronchopneumonia, intussusception, dehydration, or any other unnamed complication.

An analysis of demographics by inpatient status revealed that inpatients and outpatients were similar on most demographic characteristics, including child age, child gender, rural residence, and transportations used to arrive at the current treatments. However, inpatient families were more likely to seek previous care, to report SUMI, and had slightly larger households (p < 0.01).

Because of the substantial proportion of missing cost data (50% of observations were missing some cost data), we also analyzed demographic characteristics of those with missing cost data. Most demographic variables (e.g. child sex, age) were not associated with cost data missingness. However, those with missing cost data were significantly more likely to be inpatients (87.5% vs. 39.5%, p < 0.01), less likely to have SUMI (60.1% vs. 88%, p < 0.01), more likely to have sought previous treatment (89% vs. 76%, p = 0.01), less likely to be dual-income households (1% vs. 29%, p < 0.01), and less likely to have the participating caregiver be the child’s mother (85% vs. 92%, p < 0.01). Missingness was also associated with hospital (p < 0.01), but not with whether or not the hospital was private (p = 0.94).

### Incurred costs

To understand the economic burden that pediatric diarrhea places on Bolivian caregivers, we collected data regarding direct and indirect costs and stratified on visit type as well as hospital type (Table 3). These analyses revealed similar patterns. Inpatients, as compared to outpatients, incurred significantly (p < 0.01) higher total costs (US$45.50 vs. US$25), direct costs (US$36 vs. US$14.50), and indirect costs (US$18.50 vs. US$13), although it is worth noting that there was more missing data for indirect costs in inpatients as compared to outpatients. Similarly, patients attending the private hospital as compared to those attending public hospitals also incurred significantly (p < 0.01) higher total costs (US$59 vs. US$20), direct costs (US$43.50 vs. US$13.50), and indirect costs (US$17.50 vs. US$12.50). The majority of cost subcategories within direct medical costs were also significantly higher for inpatients as compared to outpatients and for patients at the private hospital as compared to those at public hospitals. However, while most non-medical direct costs were higher for inpatients as compared to outpatients, non-medical direct costs were mostly similar between patients at the private hospital as compared to those at public hospitals.

**Table 3:** Mean costs ($US*) for treatment incurred by the caregiver for an episode of pediatric diarrhea in a sample of 551 Bolivian children seeking care for acute diarrhea, 2007 – 2009, stratified by inpatient status and hospital type

**Type of cost**	**Inpatients**			**Outpatients**			P^**^	**Private hospital**			**Public hospital**			P^**^
	**n**	**Mean**	**SEM**	**n**	**Mean**	**SEM**		**n**	**Mean**	**SEM**	**n**	**Mean**	**SEM**	
*Direct medical costs*	297			254				203			333			
Diagnostic		2.00	0.50		1.00	0.50	*0.04*		2.50	1.00		1.00	0.50	*<0.01*
Medicines		5.00	1.00		6.50	0.50	*<0.01*		8.50	1.00		3.50	0.50	*<0.01*
Consultation Fees		2.00	0.50		19.50	2.50	*<0.01*		26.00	3.00		1.00	0.00	*<0.01*
*Total*		8.50	1.50		27.00	2.50	*<0.01*		37.00	3.50		5.50	1.00	*<0.01*
*Direct non-medical costs*	297			254				203			333			
Transportation		3.00	0.50		3.00	0.50	0.59		2.50	0.50		3.00	0.50	0.47
Food during visit		2.00	0.00		1.00	0.00	*<0.01*		1.50	0.00		1.50	0.00	0.45
Diapers		3.50	0.50		1.00	0.00	*<0.01*		1.00	0.00		3.00	0.50	*<0.01*
Child care		0.50	0.50		0.50	0.50	0.23		1.50	0.50		0.50	0.50	0.15
* Total*		9.00	0.50		5.50	0.50	*<0.01*		6.50	1.00		8.00	0.50	0.05
*Total Direct Costs*	297	36.00	2.50	254	14.50	1.50	*<0.01*	203	43.50	3.50	333	13.50	1.00	*<0.01*
*Indirect Costs (lost wages)*	124	18.50	1.50	243	13.00	1.50	*<0.01*	183	17.50	1.50	172	12.50	2.00	*<0.01*
*Total (direct and indirect) cost per episode*^*†*^	254	45.50	3.00	297	25.00	2.00	*<0.01*	203	59.00	4.00	333	20.00	1.50	*<0.01*
*Cost burden (Total Costs as % of annual income)*	254	2.2	0.2	297	1.4	0.2	*<0.01*	203	2.4	0.2	333	1.4	0.2	*<0.01*

^*^Mean costs and SEMs are rounded to the nearest $0.50. ^†^Total costs less than the sum of direct and indirect costs due to the missing values for indirect costs, which are treated as zero in total cost calculations. ^**^P-value for Kruskal-Wallis one-way analysis of variance test (non-parametric). Italicized text indicates p < 0.05.

An analysis of the cost burden (total incurred costs as a percentage of annual family income) showed that inpatient families incurred significantly higher cost burden as compared to outpatient families (mean 2.2% vs. 1.4%, p < 0.01); similarly, families at the private hospital incurred significantly higher cost burden as compared to families attending public hospitals (mean 2.4% vs. 1.4%, p < 0.01) (Table 3). A separate analysis just among families reporting lost wages found that indirect costs accounted for an average 62.3% of total incurred costs for inpatients and 40.7% of total incurred costs for outpatients (p < 0.01), and were equivalent to 1.1% and 0.8% of total annual income, respectively (p = 0.90).

**Figure 1:**
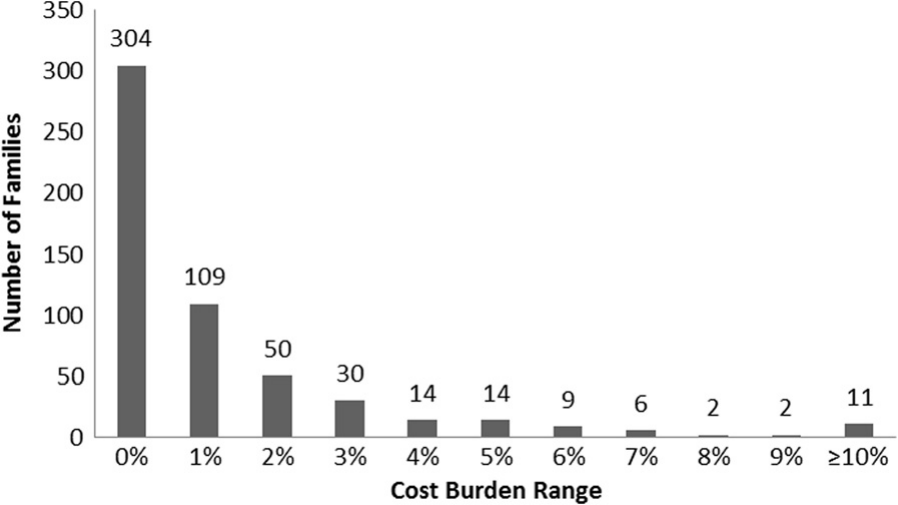
**Histogram of cost burden incurred for a single episode of pediatric diarrhea in a sample of 551 Bolivian infants seeking care for acute diarrheal illness, 2007 – 2009.** Over 40% of Bolivian families in our study spent at least 1% of their annual income on a single episode of pediatric diarrhea. The black bars represent number of patients in each cost burden category, with total number of patients at the top of the column. Each column (cost burden category) represents a range of one percentage point, for example, 0% = 0 - <1%. (N = 551).

Given that Bolivia has subsidized healthcare for pediatric populations, we also looked at differences in costs and characteristics reported by SUMI as compared to non-SUMI registered patients, though this information was available only for 57% of the study participants. Although hospital type (public vs. private) appeared to be a sensitive indicator of SUMI status (>99% of SUMI-registered patients attended a public hospital), it was not a specific indicator (52% of non-SUMI patients attended a private hospital). However, patterns of incurred costs between SUMI and non-SUMI families (Table 4) were similar to patterns by hospital type. SUMI families paid significantly less in direct medical costs as compared to non-SUMI-registered families (US$6 vs. US$25, p < 0.01), though most non-medical direct costs were similar. Indirect costs were also somewhat lower in SUMI-registered families as compared to non-SUMI families (US$14 vs. US$26.50, p < 0.01), and they also suffered a lower cost burden (1.6% vs. 1.8%, p < 0.01).Over 40% of the study population experienced a “catastrophic” cost (≥1% cost burden) (Figure 1). As expected, patients with catastrophic cost incurred higher costs as compared to those not experiencing catastrophic cost. The differences in contributing costs between patients with and without catastrophic cost included indirect costs (US$24.50 vs. US$5.50, p < 0.01), total direct costs (US$46 vs. US$7, p < 0.01), and (a component of direct costs) previous treatment costs (US$12 vs. US$2, p < 0.01).

**Table 4:** Mean costs ($US*) for treatment incurred by the caregiver for an episode of pediatric diarrhea in a sample of 551 Bolivian children seeking care for acute diarrhea, 2007 – 2009, stratified by SUMI status

**Type of cost**	**SUMI**			**Non-SUMI**			P^**^
	**n**	**Mean**	**SEM**	**n**	**Mean**	**SEM**	
*Direct medical costs*	266			47			
Diagnostic		1.00	0.50		0.50	0.00	0.42
Medicines		4.50	0.50		5.00	1.00	*<0.01*
Consultation Fees		1.00	0.00		20.00	5.50	*<0.01*
*Total*		6.00	1.00		25.00	6.00	*<0.01*
*Direct non-medical costs*	266			47			
Transportation		3.00	0.50		3.00	1.00	0.38
Food during visit		1.50	0.00		2.00	0.00	*0.01*
Diapers		2.50	0.50		4.50	1.00	0.18
Child care		0.50	0.50		0.00	0.00	0.47
* Total*		8.00	0.50		9.50	1.50	0.10
*Total Direct Costs*		14.00	1.00		34.50	6.00	*<0.01*
*Indirect Costs (lost wages)*	127	14.00	2.50	21	26.50	4.50	*<0.01*
*Total (direct and indirect) cost per episode*^*†*^	266	20.50	2.00	47	46.50	7.50	*<0.01*
*Cost burden (Total Costs as% of annual income)*	266	1.6	0.2	47	1.8	0.3	*<0.01*

^*^Mean costs and SEMs are rounded to the nearest $0.50.

^†^Total costs less than the sum of direct and indirect costs due to the missing values for indirect costs, which are treated as zero in total cost calculations.

^**^P-value for Kruskal-Wallis one-way analysis of variance test (non-parametric). Italicized text indicates p < 0.05.

### Predictors of cost burden

**Figure 2:**
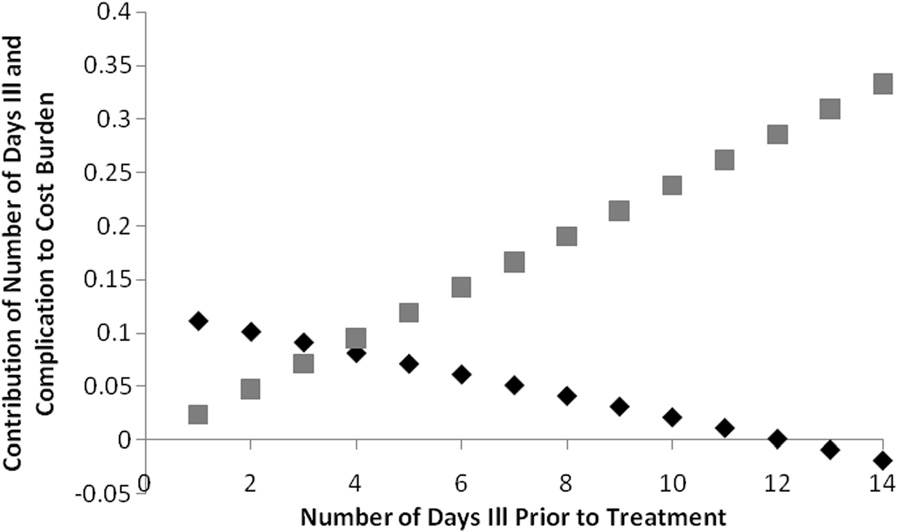
**Interaction of number of days with diarrhea and complications on cost burden incurred for a single episode of diarrhea, in a sample of 551 Bolivian infants seeking care for acute diarrheal illness, 2007–2009.** For children who present with no complications, each additional day with diarrhea prior to treatment contributes to an increasingly greater cost burden. For those children who present with at least one complication, each additional day contributes to a greater cost burden until 12 days, at which point additional days contribute to a lower cost burden. The gray squares represent the contribution of number of days with diarrhea to the log_10_ of cost burden for children presenting with no complication. The black diamonds represent the contribution of number of days with diarrhea to the log_10_ of cost burden for children presenting with at least one complication. (N = 291).

To determine potential predictors of cost burden, we constructed linear regression models with Log_10_ of cost burden as the outcome (Table 5). Bivariate analysis with the outcome, for 11 independent variables, identified seven significant associations. SUMI status was not assessed due to missing data. The adjusted R^2^ of the fully adjusted model was 0.295, and the following variables were significant (p < 0.05): private hospital (Albina Patiño vs. all others), whether the caregiver had sought treatment prior to the current visit, the number of days that the child had been ill with diarrhea prior to the current visit, outpatient status, and the interaction term between the number of days with diarrhea (1-unit change) and having at least one complication (Table 5). A sensitivity analysis using a calculation of cost burden excluding the costs of extra food and extra diapers (given that parents may have bought these items even in the absence of the child’s illness) found nearly identical results. Therefore, we proceeded with the fully inclusive cost burden when further exploring interaction. For children presenting with at least one complication, each additional day increased (by a decreasing amount, as days grew) cost burden until 12 days; additional days then lowered cost burden (Figure 2). However, for children presenting with no complications, each additional day ill contributed to an increasingly greater cost burden. Overall, we found that treatment-seeking behaviors, hospitalization, and treatment in a private hospital were important risk factors for increasing cost burden.

**Table 5:** **Linear regression model of the relationship between risk factors and the Log**_**10 **_**cost burden for one pediatric diarrheal episode, in a sample of 551 Bolivian children seeking care for acute diarrhea, 2007 - 2009**

**Variable**		**Unadjusted analysis**			**Adjusted model 1 (n = 291)**			Adjusted model 2 (n = 291)^*^		
	**N**	**β**	**Standard error**	**P**	**β**	**Standard error**	**P**	**β**	**Standard error**	**P**
*Demographics*										
Male child	544	0.136^†^	0.060	*0.02*	0.019	0.074	0.80	0.019	0.074	0.80
Age of child (months)	518	−0.006	0.003	0.08	−0.000^‡^	0.004	0.97	−0.000^‡^	0.004	0.97
Number of people in household	518	−0.000^§^	0.013	1.00	−0.010	0.016	0.53	−0.010	0.016	0.53
Rural residence	452	0.258^†^	0.088	*<0.01*	0.145	0.097	0.14	0.145	0.097	0.14
Hospital	530									
Albina Patiño (Private)		0.471^†^	0.058	*<0.01*	0.493^†^	0.073	*<0.01*	0.494^†^	0.073	*<0.01*
All Others (Public, Reference)		-	-	*-*	-	-	*-*	-	-	-
*Treatment-Seeking Behavior*										
Sought treatment at least once previously to current visit	462	0.764^†^	0.080	*<0.01*	0.438^†^	0.093	*<0.01*	0.438^†^	0.093	*<0.01*
Number of modes of transportation taken to current visit	424	0.113	0.072	0.12	0.128	0.076	0.09	0.128	0.076	0.09
Number of days child had diarrhea prior to current visit	507	0.012^†^	0.004	*<0.01*	0.024^†^	0.007	*<0.01*	0.024^†^	0.007	*<0.01*
*Severity of illness*										
Child was an outpatient	545	−0.424^†^	0.057	*<0.01*	−0.402^†^	0.109	*<0.01*	−0.402^†^	0.109	*<0.01*
Child presented with at least one complication^δ^	542	0.346^†^	0.058	*<0.01*	0.121	0.114	0.29	0.121	0.114	0.29
Child’s first episode of diarrhea	460	−0.043	0.070	0.54	0.085	0.080	0.29	0.085	0.080	0.29
*Interaction term*										
Number of days with diarrhea (1-unit change) x at least one complication		-	-	-	−0.033^†^	0.015	*0.03*^*£*^	−0.034^†^	0.015	*0.03*^*£*^

^*^Second adjusted model excludes direct non-medical costs of diapers and extra food. ^†^P < 0.05. ^‡^β = −0.0002. ^§^β = −0.00003. ^δ^Complications defined as at least one of the following, as diagnosed by the attending physician: electrolyte disorder, electrolyte imbalance, hypokalemia, metabolic acidosis, anemia, malnutrition, acute respiratory infection, bronchopneumonia, intussusception, dehydration, or any other unnamed complication. ^£^P-value associated with the overall interaction coefficient. Italicized text indicates p < 0.05.

### Predictors of catastrophic cost (Cost Burden Greater Than or Equal to 1%)

**Figure 3:**
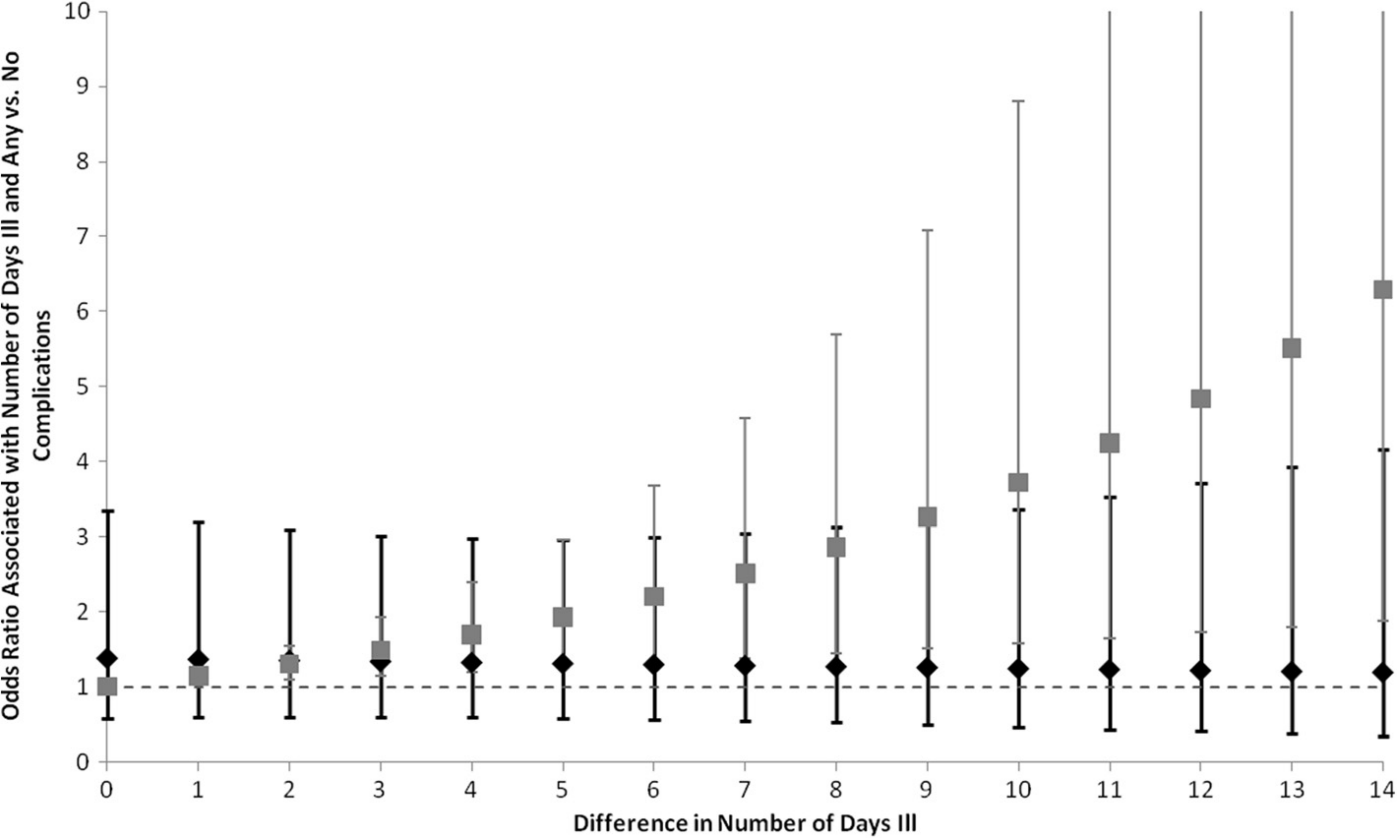
**Interaction of number of days with diarrhea and complications on risk of catastrophic cost for a single episode of diarrhea, in a sample of 551 Bolivian infants seeking care for acute diarrheal illness, 2007–2009.** The likelihood of experiencing a catastrophic cost decreases slightly as the difference in the number of days with diarrhea increases, when comparing those with a complication to those without a complication. When comparing children with no difference in complication status, the likelihood of catastrophic cost increases as the number of days with diarrhea increases. The black diamonds represent the point estimate for the odds ratio comparing children with at least one complication to those with none. The black bars are the 95% Confidence Intervals of these odds ratios. The gray squares represent the point estimate for the odds ratio associated with differences in the number of days with diarrhea, among children with no differences in their complication status. The gray bars are the 95% Confidence Intervals of these odds ratios. The dotted gray line represents an odds ratio of 1. We present odds ratios for differences in number of days with diarrhea from 0 to 14 days. (N = 295).

To identify potential predictors of catastrophic cost, we constructed logistic regression models (Table 6). Bivariate analysis with the outcome, for 11 independent variables, identified six significant associations. SUMI status was not assessed due to missing data. In the fully adjusted logistic model, we found the same significant (p < 0.05) variables as in the final linear model, with the same directions of association as well (see above). As in the linear model, a sensitivity analysis with cost calculations excluding extra food and diapers also yielded very similar results, though the interaction term was non-significant, while the measure of complications was borderline significant (p = 0.03) and in the opposite direction. We thus decided to proceed with the fully inclusive cost calculations.The odds ratio associated with the interaction term decreased with greater differences in number of days with diarrhea (comparing children with complications to those without), but was not significant between differences of one to 14 days (Figure 3). The odds ratio associated with differences in number of days with diarrhea alone increased with the number of days with diarrhea (but became less precise), and was significant throughout (p < 0.01). Similarly to results of the cost burden analysis, we found that treatment-seeking behaviors, hospitalization, and private hospital setting were important risk factors for catastrophic cost.

**Table 6:** Logistic regression model of the relationship between risk factors and catastrophic cost (cost burden ≥ 1%) for one pediatric diarrheal episode, in a sample of 551 Bolivian children seeking care for acute diarrhea, 2007 - 2009

**Variable**		**Unadjusted analysis**			**Adjusted model 1 (n = 295)**			Adjusted model 2 (n = 295)*		
	**N**	**OR**	**95% CI**	**P**	**OR**	**95% CI**	**P**	**OR**	**95% CI**	**P**
*Demographics*										
Male child	550	1.49^†^	(1.06, 2.10)	*0.02*	1.04	(0.59, 1.86)	0.89	1.00	(0.55, 1.81)	1.00
Age of child (months)	524	0.98	(0.96, 1.00)	0.07	0.99	(0.96, 1.02)	0.53	1.00	(0.96, 1.03)	0.77
Number of people in household	524	1.02	(0.95, 1.09)	0.60	1.00	(0.88, 1.13)	0.95	0.96	(0.84, 1.09)	0.51
Rural residence	458	1.76^†^	(1.09, 2.86)	*0.02*	1.54	(0.73, 3.26)	0.26	1.33	(0.62, 2.86)	0.47
Hospital	536									
Albina Patiño (Private)		3.39^†^	(2.36, 4.89)	*<0.01*	4.13^†^	(2.30, 7.41)	*<0.01*	5.26^†^	(2.86, 9.68)	*<0.01*
All Others (Public, Reference)		1.00	-	-	1.00	-	-	1.00	-	*-*
*Treatment-Seeking Behavior*										
Sought treatment at least once previously to current visit	467	8.05^†^	(3.92,16.55)	*<0.01*	3.92^†^	(1.64, 9.35)	*<0.01*	4.84^†^	(1.83,12.75)	*<0.01*
Number of transportations taken to current visit	428	1.18	(0.80, 1.75)	0.41	1.28	(0.70, 2.33)	0.42	1.79	(0.97, 3.30)	0.06
Number of days child had diarrhea prior to current visit	513	1.02	(1.00, 1.05)	0.10	1.14^†^	(1.05, 1.24)	*<0.01*	1.09^†^	(1.02, 1.16)	*0.01*
*Severity of illness*										
Child was an outpatient	551	0.35^†^	(0.25, 0.49)	*<0.01*	0.16^†^	(0.07, 0.37)	*<0.01*	0.10^†^	(0.04, 0.24)	*<0.01*
Child presented with at least one complication^‡^	548	2.10^†^	(1.49, 2.96)	*<0.01*	1.38	(0.57, 3.34)	0.47	0.42^†^	(0.19, 0.93)	*0.03*
Child’s first episode of diarrhea	466	1.1	(0.75, 1.60)	0.64	1.47	(0.79, 2.75)	0.22	1.60	(0.83, 3.06)	0.16
*Interaction term*										
Number of days with diarrhea (1-unit change) × at least one complication	-	-	-	-	1.37^†^	(0.59, 3.2)	*0.03*^*§*^	-	-	-

^*^Second adjusted model excludes direct non-medical costs of diapers and extra food. ^†^P < 0.05. ^‡^Complications were defined as at least one of the following, as diagnosed by the attending physician: electrolyte disorder, electrolyte imbalance, hypokalemia, metabolic acidosis, anemia, malnutrition, acute respiratory infection, bronchopneumonia, intussusception, dehydration, or any other unnamed complication. ^§^P-value associated with the overall interaction coefficient. Italicized text indicates p < 0.05.

## Discussion

The goals of this study were to quantify the financial impact of a single episode of pediatric diarrhea on families in Bolivia and to identify predictors of cost burden and catastrophic cost, for those seeking treatment in a hospital. The mean cost incurred for just one pediatric diarrhea episode was US$34, which could account for five to eight days of food and fuel (for cooking and heating) for an average Bolivian family with five members, depending on the family’s level of food security [39]. Nearly half the study population experienced a cost burden of over 1%, which, for a family with the median annual income of US$2,274, translates into US$22.74 spent on a single episode of diarrhea, or three to five days of food and fuel for the “average” family described above. This amount is nearly twice the average monthly expenditure on intra-city transportation for Bolivians of “moderate” poverty in 2000 [40]. In this study, cost burden increased, and the risk of catastrophic cost grew, for children who were hospitalized (versus outpatients), children whose parents had sought prior treatment for this episode of diarrhea, and children who had been sick for longer prior to the current visit. Lastly, we observed that children seen in Albina Patiño (a private hospital) showed a higher cost burden and were more likely to report a catastrophic cost, versus children seen elsewhere.

In our sample, indirect costs (lost wages) were a substantial burden on families. For families reporting lost wages, indirect costs represented an average of 62% of incurred costs for inpatients (41% for outpatients) and translated to 1.1% and 0.8% of annual income, respectively (equivalent to 3 – 4 days of income). While two studies of costs associated with pediatric diarrhea, in Canada and Britain, also found indirect costs to be important contributors to overall cost burden [18,19], most studies from LMIC settings found indirect costs to be minimal, if present [8-10,14]. Only one study, in China, showed indirect costs to be an important component of cost burden [41]. While methodology was similar (most of the studies in LMIC settings also calculated indirect costs based on caregiver reports), the study populations may have been different from our Bolivian study population [16]. In particular, we hypothesize that our study found high indirect costs because many families earned income from both spouses (30%), instead of just one. We recommend that future economic studies and cost-effectiveness analyses of the societal costs of pediatric diarrhea include indirect costs.

As significant predictors of cost burden and catastrophic cost, we identified inpatient status, previous treatment, and number of days the child had been ill with diarrhea prior to the current visit. A higher cost burden for inpatients was consistent with other literature [14,15,41], and, in Bolivia, may also reflect the higher costs associated with hospitalization versus outpatient visits (e.g. additional medications, fees, lost wages) [7]. In most other ways, inpatients were similar to outpatients (although hospitalized patients were more likely to seek previous care, to report SUMI, and had slightly larger households). The higher cost burden and risk of catastrophic cost associated with previous treatment likely reflected costs associated with that treatment, including non-medical costs as well as medical costs which may or may not have been SUMI-eligible. We believe that the measure of number of days with diarrhea captures an element of illness severity both independently and as modified by complications. Children who are sick for a long time before their parents seek hospital treatment may develop illness of a greater severity, potentially causing higher treatment costs. Additionally, these children may come from families that are less equipped or inclined to seek hospital care; perhaps parents are more fearful of incurring costs, regardless of actual ability to pay. Data was not collected on the fear of incurring costs, but we found no significant association of number of days ill with diarrhea with income per capita or monthly household income. Although rural residence was associated with cost burden in the bivariate analysis, consistent with some other studies [16], the association was no longer significant once we controlled for other variables such as hospital type and treatment-seeking behavior. Overall, these results point to the importance of treatment-seeking behavior and illness severity to overall familial cost burden.

Though few studies address the specific impact of hospital type on familial-incurred cost, we found that patients at the private hospital (Albina Patiño) had a higher cost burden and were significantly more likely to incur a catastrophic cost. This is consistent with other research. For instance, one study of pediatric diarrhea in Vietnam found indirect costs to be significantly higher in a private urban clinic, versus public and rural settings [4]; another study of illness in Indian neonates found familial expenditure to be higher for private consultations versus public (government) physicians [42]. The higher costs we observed in the private hospital are also likely related to the fact that care in private facilities in Bolivia is not covered by SUMI, Bolivia’s public insurance program for children. SUMI provides free healthcare to children under five, but only after registration (reported by 85% of our study population with this data) and only in public facilities (attended by 62%). In our study, families at the private hospital also had significantly higher average costs for previous treatments, perhaps because previous treatments also weren’t SUMI-eligible, because they had more previous visits, or because they had some preference or willingness to spend more on their child’s health. Families may elect to pay for private consultations believing that the quality of care is better [43], or for other unexplored reasons. While SUMI status and hospital type were correlated, and non-SUMI families paid significantly more in hospital fees (consult costs) overall, non-medical direct expenditures were similar between SUMI and non-SUMI families, and both presented to private and public hospitals. Overall, these data indicate a need for further investigation into why Bolivian parents take their children to private instead of public facilities, and why SUMI-registered families still incur direct costs even at public hospitals.

Several strengths and limitations of the study should be noted. One strength was the representativeness of the study results to Bolivia given the wide breadth of sites, covering multiple regions and two types of healthcare settings (outpatient and inpatient). A second strength was the consistency of the significant results between our linear and logistic models. A third strength was the care and detail taken in the gathering of cost and income data. Cost data was reported as that which the caregiver paid, regardless of whether the cost was SUMI-eligible. A sensitivity analysis excluding diapers and food (which might have been purchased anyway) from direct non-medical costs led to similar significant model results, with the exception that the interaction term in the logistic model was non-significant, while the complications term was borderline significant and in the other direction. Household income was calculated based on direct report, and we have no reason to believe that participants inflated or underreported their income. One important limitation was missing information: not all potential predictors could be assessed, with one important covariate being SUMI status, given its high percentage of missingness and our inability to confidently impute this. Similarly, patients with missing income data were necessarily excluded from analysis. However, these patients were generally similar to included patients, though they were more likely to be inpatients, non-SUMI, and single-income households. A second limitation was that we had only one private facility in our sample, making it difficult to generalize conclusions about private versus public hospitals. The study was also limited in that it was a cross-sectional assessment of financial burden; cost burden as measured may or may not correlate to future financial difficulties.

## Conclusions

In conclusion, we have shown that hospital type, treatment behavior, and appointment type were significant predictors of overall cost burden and catastrophic cost associated with pediatric diarrhea episodes in Bolivia. These costs can represent five to eight days of food for an average family, and even a cost burden of 1% can be important in a setting where the median expenditure on food and fuel amounts to over 60% of per capita daily expenditure, leaving little for other purposes [39]. We also demonstrated that indirect costs represented a substantial burden for most families. The total incurred costs of pediatric diarrhea, extrapolated to the national level, may have represented approximately US$3MM annually in societal burden during the time period prior to full implementation of the Rotarix® vaccine into the national immunization schedule [16]. This number is expected to be less in the current setting, where vaccine coverage reached ~80% as of 2011 [44].

Further research is needed in Bolivia to understand why parents are still incurring treatment costs despite accessing public hospitals (where care should be covered by SUMI), and why some elect private over public facilities. Future research should also assess the societal cost burden in the post-vaccine era in Bolivia, as this estimate of familial economic impact would be a useful way to assess the overall economic impact of the vaccine, particularly in a setting where health care costs can be difficult to estimate given subsidized care programs like SUMI. Overall, our analysis highlights the economic importance of pediatric diarrhea to Bolivian families and suggests areas of intervention to reduce cost burden and catastrophic cost associated with pediatric diarrhea.

## Abbreviations

LMIC: Lower Middle Income Countries.

## Competing interests

The authors declare that they have no competing interests.

## Authors’ contributions

RMB developed the study objectives, contributed data entry, completed data cleaning and analysis, and drafted the manuscript. ERS contributed to the design of survey instruments. ERS, MRD, and PAR participated in data entry, preliminary data analysis, and contributed to the drafting of the manuscript. MCC, BC, EC, RP, LT, CT, and RZ contributed to study design, oversight of field work, and drafting of the manuscript. VI contributed to study design, protocol, survey instruments, and contributed to the drafting of the manuscript. JSL oversaw the development of study objectives, protocol, survey instruments, data analysis, and manuscript drafting. The final version of the manuscript has been read and approved by RMB, ERS, MRD, PAR, BC, EC, RP, LT, CT, RZ, VI, and JSL. MCC passed away prior to the finalization of the manuscript.

## Pre-publication history

The pre-publication history for this paper can be accessed here:

http://www.biomedcentral.com/1471-2458/14/642/prepub
